# Two-Year Health Outcomes in Hospitalized COVID-19 Survivors in China

**DOI:** 10.1001/jamanetworkopen.2022.31790

**Published:** 2022-09-15

**Authors:** Xinyue Yang, Chao Hou, Ye Shen, Mingyang Zhang, Kejun Zhang, Fang Wang, Yuhui Liu, Xiangyu Ma, Lixia Cheng, Jun Kang, Baoman Hu, Man Wang, Ling Zeng, Yanjiang Wang, Yong He, Guoqiang Cao, Jianxin Jiang, Paul Jones, Bin Cao, Li Li

**Affiliations:** 1 Department of Respiratory Medicine, Daping Hospital, Third Military Medical University (Army Medical University), Chongqing, China; 2 Department of Rehabilitation, The Second Affiliated Hospital, Chongqing Medical University, Chongqing, China; 3 Department of Outpatient, Daping Hospital, Third Military Medical University (Army Medical University), Chongqing, China; 4 Department of Neurology and Centre for Clinical Neuroscience, Daping Hospital, Third Military Medical University (Army Medical University), Chongqing, China; 5 Wuhan Huoshenshan Hospital, Wuhan, China; 6 Department of Epidemiology, College of Preventive Medicine, Third Military Medical University (Army Medical University), Chongqing, China; 7 Department of Medical and Research Management, Daping Hospital, Third Military Medical University (Army Medical University), Chongqing, China; 8 Wuhan Taikang Tongji Hospital, Wuhan, China; 9 Department of Trauma Medical Center, Daping Hospital, State Key laboratory of Trauma, Burns, and Combined Injury, Third Military Medical University (Army Medical University), Chongqing, China; 10 Institute of Infection and Immunology, University of London, London, United Kingdom; 11 GlaxoSmithKline, Brentford, United Kingdom; 12 Department of Pulmonary and Critical Care Medicine, National Center for Respiratory Medicine, National Clinical Research Center for Respiratory Diseases, China-Japan Friendship Hospital, Beijing, China

## Abstract

**Importance:**

Relatively little is known about the persistence of symptoms in patients with COVID-19 for more than 1 year after their acute illness.

**Objective:**

To assess the health outcomes among hospitalized COVID-19 survivors over 2 years and to identify factors associated with increased risk of persistent symptoms.

**Design, Setting, and Participants:**

This was a longitudinal cohort study of patients who survived COVID-19 at 2 COVID-19–designated hospitals in Wuhan, China, from February 12 to April 10, 2020. All patients were interviewed via telephone at 1 year and 2 years after discharge. The 2-year follow-up study was conducted from March 1 to April 6, 2022. Statistical analysis was conducted from April 20 to May 5, 2022. The severity of disease was defined by World Health Organization guideline for COVID-19.

**Exposures:**

COVID-19.

**Main Outcomes and Measures:**

The main outcome was symptom changes over 2 years after hospital discharge. All patients completed a symptom questionnaire for evaluation of symptoms, along with a chronic obstructive pulmonary disease assessment test (CAT) at 1-year and 2-year follow-up visits.

**Results:**

Of 3988 COVID-19 survivors, a total of 1864 patients (median [IQR] age, 58.5 [49.0-68.0] years; 926 male patients [49.7%]) were available for both 1-year and 2-year follow-up visits. The median (IQR) time from discharge to follow-up at 2 years was 730 (719-743) days. At 2 years after hospital discharge, 370 patients (19.8%) still had symptoms, including 224 (12.0%) with persisting symptoms and 146 (7.8%) with new-onset or worsening of symptoms. The most common symptoms were fatigue, chest tightness, anxiety, dyspnea, and myalgia. Most symptoms resolved over time, but the incidence of dyspnea showed no significant change (1-year vs 2-year, 2.6% [49 patients] vs 2.0% [37 patients]). A total of 116 patients (6.2%) had CAT total scores of at least 10 at 2 years after discharge. Patients who had been admitted to the intensive care unit had higher risks of persistent symptoms (odds ratio, 2.69; 95% CI, 1.02-7.06; *P* = .04) and CAT scores of 10 or higher (odds ratio, 2.83; 95% CI, 1.21-6.66; *P* = .02).

**Conclusions and Relevance:**

In this cohort study, 2 years after hospital discharge, COVID-19 survivors had a progressive decrease in their symptom burden, but those with severe disease during hospitalization, especially those who required intensive care unit admission, had higher risks of persistent symptoms. These results are related to the original strain of the virus, and their relevance to infections with the Omicron variant is not known.

## Key Points

**Question:**

What are the 2-year health outcomes among patients hospitalized for COVID-19 in China?

**Findings:**

In this longitudinal cohort study that included 1864 patients, the most common symptoms at 2 years after SARS-CoV-2 infection were fatigue, chest tightness, anxiety, dyspnea, and myalgia, and most symptoms resolved from 1-year to 2-year follow-up, although the incidence of dyspnea showed no significant change. Patients with severe disease during hospitalization, especially those who required intensive care unit admission, had higher risks of persistent symptoms and higher chronic obstructive pulmonary disease assessment test scores.

**Meaning:**

These findings suggest that prolonged symptoms may persist in a proportion of COVID-19 survivors for 2 years after SARS-CoV-2 infection.

## Teaser

This cohort study assesses the health outcomes among hospitalized COVID-19 survivors over 2 years to identify factors associated with increased risk of persistent symptoms.

## Introduction

By May 6, 2022, the global pandemic of COVID-19 had resulted in more than 500 million confirmed cases and 6.1 million deaths.^1^ With the emergence of new SARS-CoV-2 variants of higher transmissibility (eg, Omicron and Delta), the number of confirmed cases constantly increases.^2,3^ Although most SARS-CoV-2–infected patients recover from the acute phase, some patients may experience long-lasting health problems, including physical, cognitive, and psychological sequelae, affecting their social participation and health-related quality of life.^4,5,6^ Therefore, systematic follow-up of patients with COVID-19 discharged from the hospital is necessary to identify the trajectory of symptom burden, to understand the long-term health outcomes of this disease.

Previous studies^7,8,9,10^ have indicated that a substantial proportion of COVID-19 survivors still experience problems in various health domains after hospital discharge. In a large national cohort^7^ of people with COVID-19 and contemporary and historical controls, an increased risk of incident mental health disorders (eg, anxiety disorders and depressive disorders) was found in people with COVID-19 compared with those with seasonal influenza. In an exploratory prospective cohort study^8^ involving patients who survived 1 year following intensive care unit (ICU) treatment for COVID-19, physical, mental, or cognitive symptoms were frequently reported. We previously reported^9^ that in a cohort of 2433 hospitalized COVID-19 survivors, 45.0% of patients reported at least 1 symptom 1 year after hospital discharge, and patients with severe disease had increased risk of having more symptoms. However, whether COVID-19–related symptoms may persist for a longer time is still in question. Most recently, a longitudinal follow-up study^10^ described the evolution of health and functional outcomes among COVID-19 survivors up to 2 years and found that health-related quality of life, exercise capacity, and mental health continued to improve throughout the 2 years regardless of initial disease severity. In the current study, we investigated the dynamic trajectory of symptom burden and symptom persistence of COVID-19 survivors 2 years after discharge from 2 designated hospitals.

## Methods

This study was approved by the Ethics Committee of the Daping Hospital, Army Medical University, since its medical staff worked in the COVID-19–designated Huoshenshan Hospital and Taikang Tongji Hospital during the acute phase of the pandemic in early 2020. Verbal informed consent was obtained from COVID-19 survivors or their legal guardians before the study. The Strengthening the Reporting of Observational Studies in Epidemiology (STROBE) reporting guideline was implemented.

### Study Design and Patients

This is a longitudinal cohort study involving COVID-19 survivors who were discharged from Huoshenshan Hospital and Taikang Tongji Hospital (both in Wuhan, China) between February 12 and April 10, 2020. All adult patients with laboratory-confirmed COVID-19 were screened for eligibility. The exclusion criteria included (1) those who declined to participate, (2) those unable to be contacted, and (3) those who died before the follow-up. The 2-year follow-up study was conducted from March 1 to April 6, 2022.

### Procedures

All patients were contacted in the order of their discharge date documented in their medical record. At each follow-up, patients underwent a standardized telephone interview and completed a self-reported symptom questionnaire and a chronic obstructive pulmonary disease (COPD) assessment test (CAT), which was initially designed to assess symptom burden of patients with COPD based on a modeling study of the association between CAT scores and impact of COPD on daily life and well-being.^11,12,13^ The questionnaire at 1-year follow-up was based on symptoms that had been reported by patients during hospitalization and was described in an earlier study.^9^ The questionnaire at 2-year follow-up was based on symptoms that had been reported at 1-year follow-up, as shown in eTable 1 in the Supplement. The symptoms included in the study questionnaire were graded according to a 4-point Likert scale (no problems, mild problems, moderate problems, or severe problems). Symptoms were present if at least 1 problem was rated as moderate or severe. COVID-19 survivors with long COVID-19 symptoms were defined as having at least 1 persistent or new-onset symptom related to COVID-19 that could not be explained by an alternative disease, which is consistent with the case definition of post–COVID-19 condition.^14^ For patients not responding to the telephone interview for the first time, another 2 attempts were made. On the basis of the dynamic changes of symptom number between years 1 and 2, patients were classified into 4 categories: (1) patients with at least 1 symptom at both follow-up time points were defined as having symptoms persist; (2) patients with at least 1 symptom at 1-year follow-up without symptoms at 2-year follow-up were defined as having symptom relief; (3) patients without any symptom at 1-year follow-up but with at least 1 symptom at 2-year follow-up were defined as having new-onset symptoms, which included patients who had a symptom that was reported as a mild problem at year 1, but at 2-year follow-up was reported as moderate or severe problem; and (4) patients with no symptoms at all at both follow-up time points were defined as having no symptoms.

### Data Acquisition

Collection of clinical data during hospitalization of enrolled patients has been described in our previous study of 1-year follow-up.^9^ Briefly, demographic characteristics (self-reported age, sex, and cigarette smoking) and clinical characteristics (comorbidities and symptoms) were retrieved from electronic medical records. The severity of disease was defined by World Health Organization guideline for COVID-19.^9^ Patients with severe disease were those with fever or suspected respiratory infection, plus 1 of the following conditions: respiratory rate greater than 30 breaths per minute, severe respiratory distress, or oxygen saturation as measured by pulse oximetry less than or equal to 93% on room air. We double-entered and validated all data using EpiData software version 3.1 (EpiData Association).

### Statistical Analysis

Continuous variables were presented as median (IQR), followed by Mann-Whitney *U* test, and categorical variables were presented as absolute values along with percentages, followed by the Pearson χ^2^ test or Fisher exact test when appropriate. To test the risk of bias due to patients lost to follow-up, the clinical characteristics between the enrolled patients and those lost to follow-up were compared. As an exploratory analysis, a 1:1 propensity score– matching (PSM) was further applied between these 2 subpopulations, based on age, sex, disease severity, and coexisting disorders. To identify factors associated with the risk of occurrence of at least 2 symptoms at 2-year follow-up, symptoms persisting or new-onset symptoms during follow-up, and CAT scores of at least 10, univariable logistic regression analysis was used to identify potential risk factors with *P* < .10, and then was adjusted by a stepwise (forward likelihood ratio) selection process in multivariable logistic regression model, whereas age, sex, and disease severity were forced into the model because of their importance. All tests were 2-sided, and *P* < .05 was considered significant. All statistical analyses were performed with the use of SPSS statistical package version 26.0 for Windows (IBM SPSS Statistics) and R statistical software version 4.1.1 (R Project for Statistical Computing) from April 20 to May 5, 2022.

## Results

### Patient Characteristics

**Figure 1.**
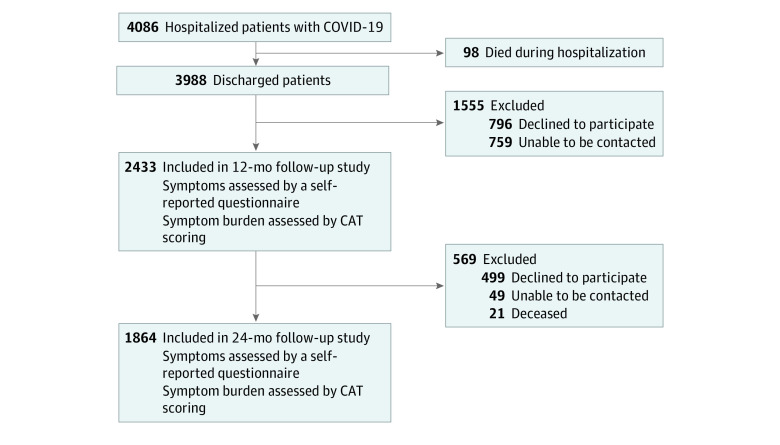
Study Flowchart CAT indicates chronic obstructive pulmonary disease assessment test.

Of 3988 COVID-19 survivors screened for eligibility, a total of 1864 patients (47.0%) who were available for both interviews were included in the final analysis; their median (IQR) age was 58.5 (49.0-68.0) years, and 926 (49.7%) were male (Figure 1). In total, 505 patients (27.1%) were categorized as having severe disease. The median (IQR) time from discharge to follow-up was 364 (357-371) days at 1 year and 730 (719-743) days at 2 years. The median (IQR) duration of hospital stay was 14 (9-20) days. During hospitalization, 1341 patients (71.9%) received oxygen therapy, among whom 14 patients (0.8%) received mechanical ventilation, and 36 patients (1.9%) were admitted to the ICU. Compared with enrolled patients, the 2124 patients lost to follow-up were older, had more coexisting disorders, including cerebrovascular diseases, hypertension, cardiovascular disease, chronic kidney diseases, and COPD, and had a higher percentage of ICU admission and mechanical ventilation. No significant difference was found in terms of sex, disease severity, the percentage of smokers, or symptoms during hospitalization (Table 1 and eTable 2 in the Supplement). Moreover, no differences in clinical characteristics or 1-year symptoms were seen between the enrolled patients and those lost to follow-up between the 1-year and 2-year interviews (eTable 3 and eTable 4 in the Supplement).

**Table 1.** Characteristics of Enrolled Participants

Characteristic	Patients, No. (%)				*P* value^a^	
	Enrolled (n = 1864)	Severe disease (n = 505)	Nonsevere disease (n = 1359)	Lost to follow-up (n = 2124)	Enrolled vs lost to follow-up	Severe vs nonsevere
Age, median (IQR), y	58.5 (49.0-68.0)	64.0 (55.0-71.0)	57.0 (46.0-66.0)	63.0 (52.0-71.0)	<.001^b^	<.001^b^
Sex,						
Male	926 (49.7)	270 (53.5)	656 (48.3)	1054 (49.6)	.97	.05
Female	938 (50.3)	235 (46.5)	703 (51.7)	1070 (50.4)		
Severe disease	505 (27.1)	NA	NA	635 (29.9)	.05	NA
Cigarette smoking						
Never	1711 (91.8)	468 (92.3)	1243 (91.5)	1969 (92.7)	.46	.70
Former	33 (1.8)	8 (1.6)	25 (1.8)	29 (1.4)		
Active	120 (6.4)	29 (5.7)	91 (6.7)	126 (5.9)		
Coexisting disorders, No.						
0	1072 (57.5)	229 (45.3)	822 (62.1)	1073 (50.5)	<.001	<.001
1	464 (24.9)	140 (27.7)	313 (23.7)	589 (27.7)		
≥2	328 (17.6)	136 (27.0)	188 (14.2)	462 (21.8)		
Type of coexisting disorder						
Hypertension	544 (29.2)	206 (40.8)	338 (24.9)	696 (32.8)	.02	<.001
Diabetes	259 (13.9)	99 (19.6)	160 (11.8)	308 (14.5)	.58	<.001
Cardiovascular diseases	164 (8.8)	67 (13.3)	97 (7.1)	247 (11.6)	.003	<.001
Chronic liver disease	96 (5.2)	24 (4.8)	72 (5.3)	116 (5.5)	.66	.64
Cerebrovascular disease	47 (2.5)	19 (3.8)	28 (2.1)	128 (6.0)	<.001	.04
Chronic kidney disease	38 (2.0)	13 (2.6)	25 (1.8)	68 (3.2)	.02	.32
Tumor	32 (1.7)	13 (2.6)	19 (1.4)	55 (2.6)	.06	.08
Tracheitis	30 (1.6)	13 (2.6)	17 (1.3)	48 (2.3)	.14	.05
Chronic obstructive pulmonary disease	16 (0.9)	7 (1.4)	9 (0.7)	35 (1.6)	.03	.16^c^
Length of hospital stay, median (IQR), d	14 (9-20)	15 (10-23)	14 (9-20)	14 (9-20)	.60	<.001^a^
Intensive care unit admission	36 (1.9)	30 (5.9)	6 (0.4)	72 (3.4)	.005	<.001
Oxygen therapy	1341 (71.9)	447 (88.5)	894 (65.8)	1559 (73.4)	.30	<.001
Mechanical ventilation	14 (0.8)	13 (2.6)	1 (0.1)	33 (1.6)	.02	<.001^c^

Abbreviation: NA, not applicable.

^a^ Statistical tests were calculated with the Pearson χ^2^ test unless otherwise noted.

^b^ Calculated with Mann-Whitney *U* test.

^c^ Calculated with Fisher exact test.

### Characteristics of Long-term Symptoms at 1-Year and 2-Year Follow-up

During hospitalization, 1777 patients (95.3%) were found with at least 1 COVID-19–related symptom. During follow-up, the proportion of patients with long COVID-19 symptoms constantly decreased (1-year vs 2-year, 43.2% [806 patients] vs 19.8% [370 patients]; difference, 23.4%; 95% CI, 20.5%-26.3%; *P* < .001). The decrease was seen in patients with severe and nonsevere disease and was numerically greater in those with severe disease (severe disease, 1-year vs 2-year, 52.3% [264 patients] vs 24.4% [123 patients]; difference, 27.9%; 95% CI, 22.2%-33.7%; *P* < .001; nonsevere disease, 1-year vs 2-year, 39.9% [542 patients] vs 18.2% [247 patients]; difference, 21.7%; 95% CI, 18.4%-25.0%; *P* < .001). Of those with symptoms at 2-year follow-up, 228 patients (12.2%) reported 1 symptom, 83 (4.5%) reported 2 symptoms, and 59 (3.2%) reported 3 or more symptoms (eFigure 1 in the Supplement).

**Figure 2.**
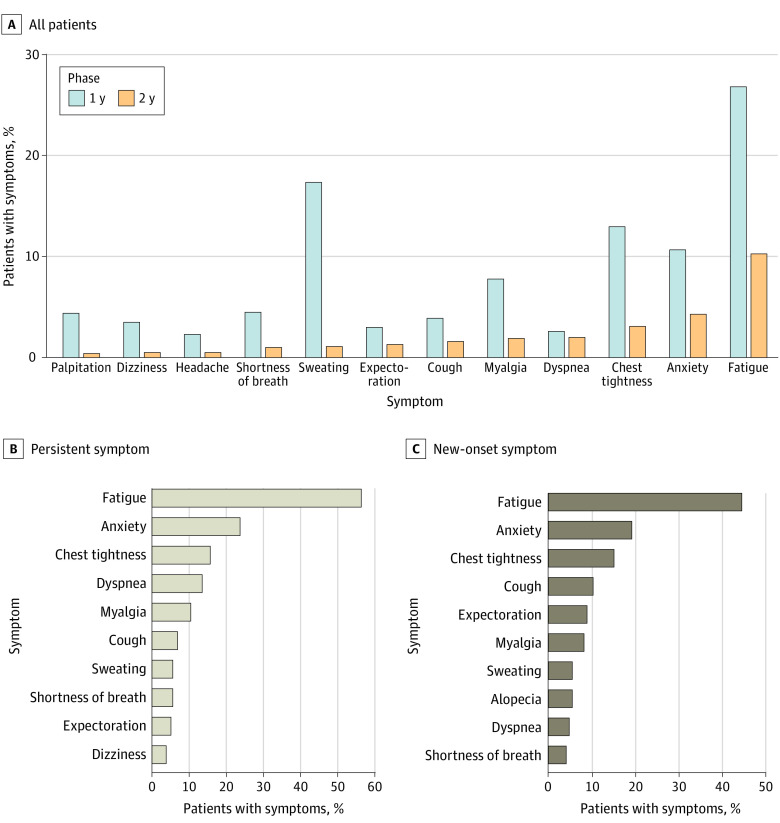
Symptoms of COVID-19 Survivors During 2-Year Follow-up A, Percentage of patients presenting with COVID-19–related symptoms at 1-year and 2-year follow-up. B, Proportion of different symptoms in the symptoms persist group and new-onset symptoms group at 2-year follow-up.

At 1 year after discharge, the most common symptoms among COVID-19 survivors were fatigue, sweating, chest tightness, anxiety, and myalgia, whereas at 2-year follow-up, the most common symptoms were fatigue, chest tightness, anxiety, dyspnea, and myalgia (Figure 2A; full details are shown in eTable 5 in the Supplement). Fatigue was the most commonly reported symptom at both interviews, whereas the proportion decreased from 26.9% (501 of 1864 patients) at 1-year follow-up to 10.3% (192 of 1864 patients) at 2-year follow-up. Most other symptoms also significantly resolved over time, yet a small percentage of patients reported dyspnea and it showed no significant change (1-year vs 2-year, 2.6% [49 patients] vs 2.0% [37 patients]).

After PSM, 1691 patients (90.7%) in the enrolled population were matched successfully with those lost to follow-up, and the baseline characteristics were comparable (eTable 6 in the Supplement). In the PSM population, the most common symptoms at 2-year follow-up were fatigue (178 patients [10.5%]), anxiety (74 patients [4.4%]), chest tightness (47 patients [2.8%]), dyspnea (35 patients [2.1%]), and myalgia (32 patients [1.9%]), which were similar to those for the overall enrolled population (eTable 7 and eTable 8 in the Supplement).

### Dynamic Trajectory of Symptoms During 2-Year Follow-up

Regarding the symptom dynamics over 2 years, 224 patients (12.0%) were classified as having symptoms persist, 582 (31.2%) as experiencing symptom relief, 146 (7.8%) as having new-onset symptoms, and 912 (48.9%) as having no symptoms (Table 2). Patients with severe disease were more likely to be classified as having symptoms persist (severe vs nonsevere, 17.6% [89 patients] vs 9.9% [135 patients] at 2-year follow-up) and were less likely to be classified as having no symptoms (severe vs nonsevere, 41.0% [207 patients] vs 51.9% [705 patients]). Similarly, in the PSM population, patients with severe disease were more likely to be classified as having symptoms persist, whereas patients with nonsevere disease were more likely to be classified as having no symptoms (eTable 9 in the Supplement). Fatigue, anxiety, chest tightness, dyspnea, and myalgia were the most common symptoms in the symptoms persist group. On the other hand, the most common symptoms in the new-onset symptoms group were fatigue, anxiety, chest tightness, cough, and expectoration (Figure 2B). Of note, the proportion of dyspnea was much higher in the symptoms persist group than the new-onset symptoms group (30 patients [13.4%] vs 7 patients [4.8%]).

**Table 2.** Symptom Dynamics of COVID-19 Survivors According to Disease Severity

Categories	No. of symptoms		Patients, No. (%)			*P* value, severe vs nonsevere disease
	1-y follow-up	2-y follow-up	Enrolled patients	Severe disease	Nonsevere disease	
Total	NA	NA	1864 (100.0)	505 (100.0)	1359 (100.0)	NA
Symptoms persist^a^	≥1	≥1	224 (12.0)	89 (17.6)	135 (9.9)	<.001
Symptom relief^b^	≥1	0	582 (31.2)	175 (34.7)	407 (29.9)	.05
New-onset symptoms^c^	0	≥1	146 (7.8)	34 (6.7)	112 (8.2)	.28
No symptoms^d^	0	0	912 (48.9)	207 (41.0)	705 (51.9)	<.001

Abbreviation: NA, not applicable.

^a^ Refers to patients with at least 1 symptom at both 1-year and 2-year follow-up.

^b^ Refers to patients with at least 1 symptom at 1-year follow-up but without any symptoms at 2-year follow-up.

^c^ Refers to patients without any symptoms at 1-year follow-up, but reported at least 1 symptom at 2-year follow-up, including patients who had a symptom that was reported as a mild problem at year 1, but at 2-year follow-up was reported as moderate or severe problem.

^d^ Refers to patients with no symptoms at any follow-up.

### Factors Associated with Risk of Symptom Persistence at 2-Year Follow-up

On univariable analysis, age, sex, disease severity, and ICU admission were associated with symptom persistence, compared with the symptom relief group. In a multivariable analysis, only ICU admission (odds ratio [OR], 2.69; 95% CI, 1.02-7.06; *P* = .04) was associated with a higher risk of symptom persistence at 2 years (eTable 10 in the Supplement). Coexisting cerebrovascular diseases (OR, 3.23; 95% CI, 1.36-7.69; *P* = .008) were associated with new-onset symptoms, compared with the no symptoms group (eTable 11 in the Supplement). Of note, of 47 patients with coexisting cerebrovascular diseases, 43 (91.5%) had more than 2 coexisting diseases, which was much higher than the proportion among patients with coexisting diseases other than cerebrovascular diseases (285 of 745 patients [38.3%]). In patients who had more than 2 symptoms at 2-year follow-up, age (OR, 1.03; 95% CI, 1.01-1.04; *P* < .001) and chronic liver disease (OR, 2.22; 95% CI, 1.19-4.14; *P* = .01) were independently associated with the risk of symptom persistence (eTable 12 in the Supplement).

### CAT Scores at 2-Year Follow-up

**Figure 3.**
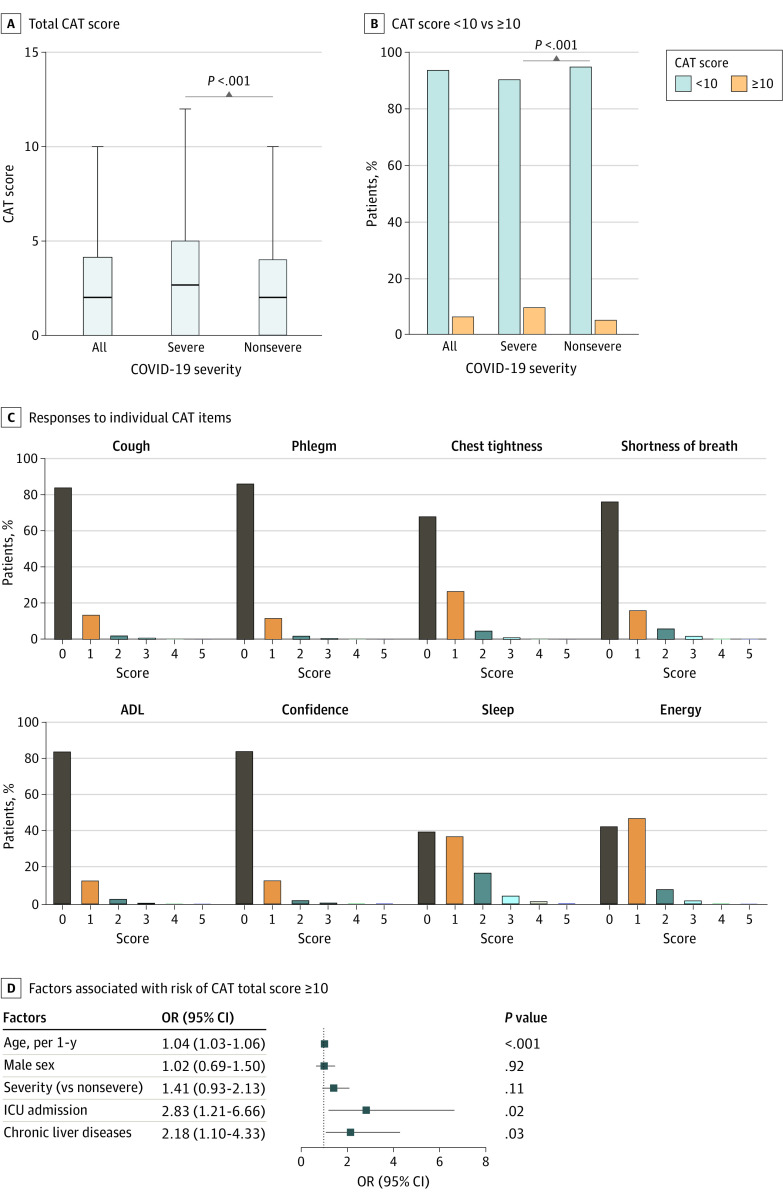
Total and Item Chronic Obstructive Pulmonary Disease (COPD) Assessment Test (CAT) Scores in Patients at 2-Year Follow-up A, Total CAT score at 2 years after patient discharge; lines within bars denote means, and error bars denote 95% CIs. B, Percentage of patients with CAT score 10 or higher and less than 10. C, Histogram of responses to individual CAT items; confidence refers to a CAT item concerning confidence leaving the home. D, Factors associated with risk of CAT total scores 10 or higher. ADL indicates activities of daily living; OR, odds ratio.

Previously, CAT has been used to assess symptom burden of patients with COVID-19.^9,15^ At 2 years after hospital discharge, the median (IQR) CAT score was 2 (0-4) in the total cohort, and the severe disease group had a significantly higher median (IQR) CAT score compared with the nonsevere group (3 [0-5] vs 2 [0-4]) (Figure 3A). A total of 116 patients (6.2%) had CAT total scores of 10 or higher, and the proportion was higher in the severe disease group than in the nonsevere disease group (severe vs nonsevere, 48 patients [9.5%] vs 68 patients [5.0%]) (Figure 3B). Patients who had CAT total scores of 10 or more were older, had a higher proportion of severe disease, more coexisting disorders, longer length of hospital stays, and a greater use of oxygen therapy than patients with lower CAT scores (eTable 13 in the Supplement).

The CAT item scores are shown in Figure 3C, which suggest that more patients tended to have sleep disorder and poor energy state than other symptoms. After multivariable adjustment, age (OR, 1.04; 95% CI, 1.03-1.06; *P* < .001), ICU admission (OR, 2.83; 95% CI, 1.21-6.66; *P* = .02), and chronic liver disease (OR, 2.18; 95% CI, 1.10-4.33; *P* = .03) were found to be factors independently associated with the risk of CAT scores of 10 or higher at 2-year follow-up (Figure 3D and eTable 14 in the Supplement). Higher CAT item scores regarding breathlessness, sleep, and energy were found in those with chronic liver disease (eFigure 2 in the Supplement).

## Discussion

In this cohort study, at 2 years after hospital discharge, 370 patients (19.8%) still had symptoms, including 224 (12.0%) with persistent symptoms and 146 (7.8%) reporting new-onset or worsening symptoms from a reported level of mild at year 1. The most common symptoms were fatigue, chest tightness, anxiety, dyspnea, and myalgia. Most symptoms resolved over time, except for dyspnea, but at 1 year it was already at a low level. ICU admission was associated with higher risks of symptoms persisting, whereas coexisting cerebrovascular diseases were associated with new-onset symptoms. In total, 116 patients (6.2%) had CAT total scores of at least 10, for whom the factors associated with increased risk included ICU admission during hospital stay or coexisting chronic liver diseases. Taken together, these findings add to our current knowledge of health outcome dynamics of COVID-19.

For 2-year survivors of COVID-19, the most common symptom was fatigue, which decreased from 26.9% at 1-year follow-up to 10.3% at 2-year follow-up. There was a general decreasing trend for this symptom, which was confirmed in another study.^16^The post–COVID-19 fatigue is similar to postinfectious fatigue syndromes following other well-documented infectious diseases,^17^ including SARS-CoV-1 (the cause of severe acute respiratory disease)^18^ and Ebola virus,^19^ among others. For SARS-CoV-1, postinfectious fatigue could last as long as 4 years.^20^

Most patients with dyspnea at 1-year follow-up still had this symptom at 2-year follow-up. Previously, Bellan et al^21^ observed that the proportion of patients with COVID-19 with dyspnea increased significantly over time, despite a stable diffusing capacity for carbon monoxide. In another prospective, longitudinal study^22^ of patients with SARS-CoV-1, at 24 months after disease onset, persistent impairment of 6-m walk distance was observed, and it contributed to the reduced quality of life. The current study identified chronic liver disease as a factor associated with the risk of symptom persistence, as well as CAT scores of 10 or higher. Previously, 1 large multicenter study^23^ identified specific subgroups of patients with chronic liver disease who had higher mortality with COVID-19. In the current study, patients with chronic liver disease had higher item scores regarding breathlessness, sleep, and energy, which indicates that coexisting chronic liver disease may be associated with both pulmonary-specific and nonspecific CAT subitems.

The presence of patients with new-onset symptoms raises important questions about causality and the mechanisms that could result in the development of symptoms at least 1 year after the acute illness. Our definition of new-onset symptoms included patients who had a symptom that was reported as mild at year 1 but moderate or severe at year 2. This could include patients who initially had a mild symptom that worsened over time because of a progressive element to their disease. Coexisting cerebrovascular diseases were associated with increased risk of new-onset symptoms, and it has been reported that coexisting cerebrovascular disease during hospitalization was 1 of the top 3 factors associated with COVID-19 severity^24^ and was associated with postacute sequelae of COVID-19.^25^ We also found that patients with coexisting cerebrovascular disease had more coexisting disorders of other organ systems, which raised the possibility that diseases other than COVID-19 may have been associated with the new-onset symptoms, so it is difficult to determine whether new-onset symptoms were completely attributable to long COVID-19.

### Limitations

There are several limitations to our study. The first is common to most studies of COVID-19: the absence of an age-matched and comorbidity-matched control group. It is, therefore, not possible to directly ascribe the patients’ long-term symptoms to the acute illness, particularly for patients who are at an age when comorbidities and their associated symptoms are common and will increase over time. For example in a population study in Australia,^26^ 9.5% of people surveyed had a modified Medical Research Council dyspnea score of 2 or higher. However, the longitudinal nature of our study showing progressive reduction in symptoms over time following the acute episode suggests an association between the acute event and the persistent symptoms. In terms of limitations that are specific to this study, the enrolled patients were less than half of the eligible population discharged from hospital. Patients lost to follow-up were older than those who continued in the study, which is important because age is an effect modifier of post–COVID-19 symptoms, and older patients had more pre-existing disorders, thus introducing a risk of survivor bias. Although we performed a PSM process, this method is limited to the factors that were measured, and other important unmeasured factors may have been operating, so residual selection bias may have persisted. Second, we used a self-reported symptom questionnaire rather than specific diagnostic tools, predisposing a risk of bias due to patients’ subjectivity, and the number of symptoms involved in our study was small considering that more than 100 potential COVID-19–related symptoms have been reported,^27,28^ which may introduce bias in that patients are less likely to willingly provide information not on the survey questionnaires.^29,30^ Third, constantly emerging coronavirus variants have been endemic^31^ and may have different virulence and long-term sequelae with our findings.

## Conclusions

In this longitudinal cohort study that included 1864 hospitalized COVID-19 survivors, the most common symptoms at 2 years after discharge were fatigue, chest tightness, anxiety, dyspnea, and myalgia. Most symptoms resolved, yet dyspnea persisted at a very low level over time. Patients with severe disease during hospitalization, especially ICU admission, had higher risks of symptom persistence and CAT total scores of at least 10. The findings provide valuable information about the dynamic trajectory of long-term health outcomes of COVID-19 survivors.

## Notes

Supplement. **eTable 1.** Symptom Questionnaire **eTable 2.** Symptoms During COVID-19 Hospitalization of Enrolled Patients and Those Lost to Follow-up **eTable 3.** Characteristics of Enrolled Participants and Those Lost to Follow-up Between 1-Year and 2-Year Follow-up **eTable 4.** One-Year Sequelae of Enrolled Participants and Those Lost to Follow-up Between 1-Year and 2-Year Follow-up **eFigure 1.** Percentage of Patients Presenting With Different Numbers of Symptoms at 1-Year Follow-up and 2-Year Follow-up **eTable 5.** Dynamic Changes of Symptoms During 2-Year Follow-up According to Disease Severity **eTable 6.** Characteristics of the Propensity Score-Matched Population **eTable 7.** Symptoms at 1-Year Follow-up and 2-Year Follow-up in Overall and Propensity Score-Matched Populations **eTable 8.** Symptoms at 2-Year Follow-up in Overall and Propensity Score-Matched Populations According to Disease Severity **eTable 9.** Symptom Dynamics of COVID-19 Survivors According to Disease Severity in Propensity Score-Matched Population **eTable 10.** Logistic Regression Models to Evaluate the Risk Factors for Symptom Persist vs Relief **eTable 11.** Logistic Regression Models to Evaluate the Risk Factors for Symptom New-Onset vs No Sequelae **eTable 12.** Logistic Regression Models to Evaluate the Risk Factors for More Than Two Symptoms at 2-Year Follow-up **eTable 13.** Patient Characteristics Between Those With CAT Total Scores ≥ 10 and Those < 10 **eTable 14.** Logistic Regression Models to Evaluate the Risk Factors for CAT Score ≥10 **eFigure 2.** CAT Item Scores Between Patients With and Without Coexisting Chronic Liver Disease
