# Supplementary material for: Two-Year Health Outcomes in Hospitalized COVID-19 Survivors in China
Source: JAMA Netw Open. 2022 Sep 15;5(9):e2231790. doi: 10.1001/jamanetworkopen.2022.31790 (PMC9478774; doi:10.1001/jamanetworkopen.2022.31790)
Supplement: Supplement. — eTable 1. Symptom Questionnaire eTable 2. Symptoms During COVID-19 Hospitalization of Enrolled Patients and Those Lost to Follow-up eTable 3. Characteristics of Enrolled Participants and Those Lost to Follow-up Between 1-Year and 2-Year Follow-up eTable 4. One-Year Sequelae of Enrolled Participants and Those Lost to Follow-up Between 1-Year and 2-Year Follow-up eFigure 1. Percentage of Patients Presenting With Different Numbers of Symptoms at 1-Year Follow-up and 2-Year Follow-up eTable 5. Dynamic Changes of Symptoms During 2-Year Follow-up According to Disease Severity eTable 6. Characteristics of the Propensity Score-Matched Population eTable 7. Symptoms at 1-Year Follow-up and 2-Year Follow-up in Overall and Propensity Score-Matched Populations eTable 8. Symptoms at 2-Year Follow-up in Overall and Propensity Score-Matched Populations According to Disease Severity eTable 9. Symptom Dynamics of COVID-19 Survivors According to Disease Severity in Propensity Score-Matched Population eTable 10. Logistic Regression Models to Evaluate the Risk Factors for Symptom Persist vs Relief eTable 11. Logistic Regression Models to Evaluate the Risk Factors for Symptom New-Onset vs No Sequelae eTable 12. Logistic Regression Models to Evaluate the Risk Factors for More Than Two Symptoms at 2-Year Follow-up eTable 13. Patient Characteristics Between Those With CAT Total Scores ≥ 10 and Those < 10 eTable 14. Logistic Regression Models to Evaluate the Risk Factors for CAT Score ≥10 eFigure 2. CAT Item Scores Between Patients With and Without Coexisting Chronic Liver Disease [file jamanetwopen-e2231790-s001.pdf]

Supplemental Online Content

Yang X, Hou C, Shen Y, et al. Two-year health outcomes in hospitalized COVID-19 survivors in China. *JAMA Netw Open*. 2022;5(9):e2231790. doi:10.1001/jamanetworkopen.2022.31790

eTable 1. Symptom Questionnaire
eTable 2. Symptoms During COVID-19 Hospitalization of Enrolled Patients and Those Lost to Follow-up
eTable 3. Characteristics of Enrolled Participants and Those Lost to Follow-up Between 1-Year and 2-Year Follow-up
eTable 4. One-Year Sequelae of Enrolled Participants and Those Lost to Follow-up Between 1-Year and 2-Year Follow-up
eFigure 1. Percentage of Patients Presenting With Different Numbers of Symptoms at 1-Year Follow-up and 2-Year Follow-up
eTable 5. Dynamic Changes of Symptoms During 2-Year Follow-up According to Disease Severity
eTable 6. Characteristics of the Propensity Score-Matched Population
eTable 7. Symptoms at 1-Year Follow-up and 2-Year Follow-up in Overall and Propensity Score-Matched Populations
eTable 8. Symptoms at 2-Year Follow-up in Overall and Propensity Score-Matched Populations According to Disease Severity
eTable 9. Symptom Dynamics of COVID-19 Survivors According to Disease Severity in Propensity Score-Matched Population
eTable 10. Logistic Regression Models to Evaluate the Risk Factors for Symptom Persist vs Relief
eTable 11. Logistic Regression Models to Evaluate the Risk Factors for Symptom New-Onset vs No Sequelae
eTable 12. Logistic Regression Models to Evaluate the Risk Factors for More Than Two Symptoms at 2-Year Follow-up
eTable 13. Patient Characteristics Between Those With CAT Total Scores ≥ 10 and Those < 10
eTable 14. Logistic Regression Models to Evaluate the Risk Factors for CAT Score ≥10
eFigure 2. CAT Item Scores Between Patients With and Without Coexisting Chronic Liver Disease

This supplemental material has been provided by the authors to give readers additional information about their work.

© 2022 Yang X et al. *JAMA Network Open.*

eTable 1. Symptom Questionnaire

Number: ____ Name: ____ Willingness to participate: ☐yes ☐no ☐lost-to-follow up

1.Dyspnea: ☐no problems ☐mild problems ☐moderate problems ☐severe problems
2.Cough: ☐no problems ☐mild problems ☐moderate problems ☐severe problems
3.Expectoration: ☐no problems ☐mild problems ☐moderate problems ☐severe problems
4.Hemoptysis: ☐no problems ☐mild problems ☐moderate problems ☐severe problems
5.Sore throat: ☐no problems ☐mild problems ☐moderate problems ☐severe problems
6. Anorexia: ☐no problems ☐mild problems ☐moderate problems ☐severe problems
7.Chill: ☐no problems ☐mild problems ☐moderate problems ☐severe problems
8.Edema of lower limbs: ☐no problems ☐mild problems ☐moderate problems ☐severe problems
9.Chest tightness: ☐no problems ☐mild problems ☐moderate problems ☐severe problems
10.Short of breath: ☐no problems ☐mild problems ☐moderate problems ☐severe problems
11.Palpitation: ☐no problems ☐mild problems ☐moderate problems ☐severe problems
12.Dizziness: ☐no problems ☐mild problems ☐moderate problems ☐severe problems
13.Headache: ☐no problems ☐mild problems ☐moderate problems ☐severe problems
14.Fatigue: ☐no problems ☐mild problems ☐moderate problems ☐severe problems
15.Myalgia: ☐no problems ☐mild problems ☐moderate problems ☐severe problems
16.Anxiety: ☐no problems ☐mild problems ☐moderate problems ☐severe problems
17.Sweating: ☐no problems ☐mild problems ☐moderate problems ☐severe problems
18.Smell reduction: ☐no problems ☐mild problems ☐moderate problems ☐severe problems
19.Taste change: ☐no problems ☐mild problems ☐moderate problems ☐severe problems
20.Diarrhea: ☐no problems ☐mild problems ☐moderate problems ☐severe problems
21.Nausea: ☐no problems ☐mild problems ☐moderate problems ☐severe problems
22.Vomiting: ☐no problems ☐mild problems ☐moderate problems ☐severe problems
Other COVID-19-related symptoms, if any:

© 2022 Yang X et al. *JAMA Network Open.*

eTable 2. Symptoms During COVID-19 Hospitalization of Enrolled Patients and Those Lost to Follow-up

Symptoms	Enrolled patients (n=1864)	Severe (n=505)	Non-severe (n=1359)	Lost to follow-up (n=2124)	*P* value Enrolled patients vs. Lost to follow-up	*P* value Severe vs. Non-Severe
Anyone of the following symptoms	1777(95.3)	486(96.2)	1291(95.0)	2012(94.7)	.38	.26
Fever	1464(78.5)	416(82.4)	1048(77.1)	1630(76.7)	.17	.01
Cough	1313(70.4)	377(74.7)	936(68.9)	1453(68.4)	.17	.02
Fatigue	1037(55.6)	302(59.8)	735(54.1)	1143(53.8)	.25	.03
Anorexia	948(50.9)	264(52.3)	684(50.3)	1143(53.8)	.06	.46
Short of breath	781(41.9)	272(53.9)	509(37.5)	847(39.9)	.20	<0.001
Chest tightness	540(29.0)	181(35.8)	359(26.4)	608(28.6)	.81	<0.001
Myalgia	502(26.9)	138(27.3)	364(26.8)	570(26.8)	.95	.82
Expectoration	312(16.7)	100(19.8)	212(15.6)	401(18.9)	.08	.03
Dyspnea	137(7.3)	81(16.0)	56(4.1)	152(7.2)	.81	<0.001
Diarrhea	129(6.9)	31(6.1)	98(7.2)	136(6.4)	.51	.42
Sore throat	110(5.9)	24(4.8)	86(6.3)	112(5.3)	.39	.20
Nausea	56(3.0)	14(2.8)	42(3.1)	60(2.8)	.73	.72
Vomiting	48(2.6)	14(2.8)	34(2.5)	53(2.5)	.87	.74
Headache	46(2.5)	7(1.4)	39(2.9)	58(2.7)	.60	.07
Chill	43(2.3)	21(4.2)	22(1.6)	51(2.4)	.85	.001
Dizziness	47(2.5)	14(2.8)	33(2.4)	58(2.7)	.68	.67
Hemoptysis	14(0.8)	4(0.8)	10(0.7)	11(0.5)	.35	.90

© 2022 Yang X et al. *JAMA Network Open.*

eTable 3. Characteristics of Enrolled Participants and Those Lost to Follow-up Between 1-Year and 2-Year Follow-up

Characteristic	Enrolled patients (n=1864)	Lost to follow-up (n=569)	*P* value Enrolled patients vs. Lost to follow-up
Age-Median (IQR), years[a]	58.5(49.0-68.0)	63.0(50.5-71.0)	<.001
Sex-No. (%)			
Male	926(49.7)	279(49.0)	.79
Female	938(50.3)	290(51.0)	
Severe disease-No. (%)	505(27.1)	175(30.8)	.09
Cigarette smoking-No. (%)			.46
Never	1711(91.8)	528(92.7)	
Former	33(1.8)	6(1.1)	
Active	120(6.4)	35(6.2)	
Coexisting disorders-No. (%)			
Coexisting disorders number			<.001
0	1072(57.5)	306(53.8)	
1	464(24.9)	147(25.8)	
≥2	328(17.6)	116(20.4)	
Hypertension	544(29.2)	168(29.5)	.88
Diabetes mellitus	259(13.9)	78(13.7)	.91
Cardiovascular diseases	164(8.8)	59(10.4)	.26
Chronic liver diseases	96(5.2)	26(4.6)	.58
Cerebrovascular diseases	47(2.5)	35(6.2)	<.001
Chronic kidney diseases	38(2.0)	20(3.5)	.04
Tumor	32(1.7)	12(2.1)	.54
Tracheitis	30(1.6)	10(1.8)	.81
COPD	16(0.9)	7(1.2)	.42
Length of hospital stay, days [a]	14.0(9.0-20.0)	14.0(9.0-21.0)	.71
ICU admission-No. (%)	36(1.9)	18(3.2)	.08
Oxygen therapy, No. (%)	1341(71.9)	402(70.7)	.56
Mechanical Ventilation, No. (%)	14(0.8)	7(1.2)	.28

Abbreviation: IQR, Inter-quartile range; ICU, Intensive Care Unit; COPD, Chronic obstructive pulmonary disease; NA, not applicable.

a, Mann-Whitney *U* test. The rest of the statistical test were calculated with the Pearson χ^2 test.

© 2022 Yang X et al. *JAMA Network Open.*

eTable 4. One-Year Sequelae of Enrolled Participants and Those Lost to Follow-up Between 1-Year and 2-Year Follow-up

**Symptoms**	**Enrolled patients (n=1864)**	**Severe (n=505)**	**Non-severe (n=1359)**	**Lost to follow-up (n=569)**	***P* value Enrolled patients vs. Lost to follow-up**
Any one of the following symptoms	806(43.2)	264(52.3)	542(39.9)	289(50.8)	.002
Fatigue	501(26.9)	174(34.5)	327(24.1)	195(34.3)	.001
Sweating	324(17.4)	116(23.0)	208(15.3)	100(17.6)	.92
Chest tightness	243(13.0)	96(19.0)	147(10.8)	83(14.6)	.34
Anxiety	199(10.7)	62(12.3)	137(10.1)	63(11.1)	.79
Myalgia	145(7.8)	55(10.9)	90(6.6)	53(9.3)	.24
Palpitation	82(4.4)	29(5.7)	53(3.9)	24(4.2)	.85
Cough	72(3.9)	33(6.5)	39(2.9)	32(5.6)	.07
Short of breath	84(4.5)	34(6.7)	50(3.7)	19(3.3)	.23
Dizziness	66(3.5)	20(4.0)	46(3.4)	16(2.8)	.40
Expectoration	56(3.0)	25(5.0)	31(2.3)	19(3.3)	.69
Dyspnea	49(2.6)	23(4.6)	26(1.9)	20(3.5)	.27
Headache	42(2.3)	15(3.0)	27(2.0)	15(2.6)	.60
Edema of lower limbs	28(1.5)	13(2.6)	15(1.1)	8(1.4)	.87
Taste change	26(1.4)	10(2.0)	16(1.2)	9(1.6)	.74
Smell reduction	25(1.3)	12(2.4)	13(1.0)	7(1.2)	.84
Sore throat	18(1.0)	10(2.0)	8(0.6)	7(1.2)	.58
Anorexia	13(0.7)	3(0.6)	10(0.7)	7(1.2)	.22
Diarrhea	13(0.7)	4(0.8)	9(0.7)	5(0.9)	.66
Hemoptysis	2(0.1)	0	2(0.1)	3(0.5)	.09[a]

© 2022 Yang X et al. *JAMA Network Open.*

Nausea	3(0.2)	0	3(0.2)	2(0.4)	.33[a]
Chill	2(0.1)	0	2(0.1)	1(0.2)	.55[a]
Vomiting	1(0.1)	0	1(0.1)	2(0.4)	.14[a]

a, Fisher's exact test. The rest of the statistical test were calculated with the Pearson χ^2 test.

© 2022 Yang X et al. *JAMA Network Open.*

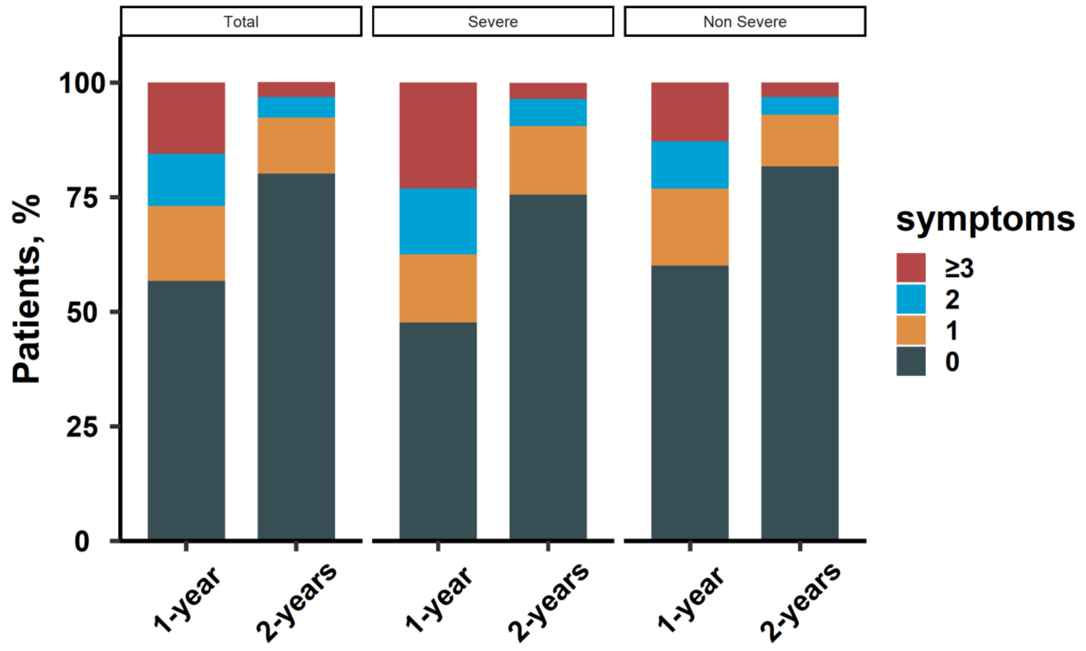

eFigure 1. Percentage of Patients Presenting With Different Numbers of Symptoms at 1-Year Follow-up and 2-Year Follow-up

© 2022 Yang X et al. *JAMA Network Open.*

eTable 5. Dynamic Changes of Symptoms During 2-Year Follow-up According to Disease Severity

Symptoms		Enrolled patients, No. (%) (n=1864)				Severe, No. (%) (n=505)				Non-severe No. (%) (n=1359)			P value Severe vs. Non-severe at 2-year
	acute	1-year	2-year	*P* value [b]	acute	1-year	2-year	*P* value [b]	acute	1-year	2-year	*P* value [b]	
Any one of the following symptoms	1777(95.3)	806(43.2)	370(19.8)	<.001	487(96.4)	264(52.3)	123(24.4)	<.001	1290(94.9)	542(39.9)	247(18.2)	<.001	.003
Fatigue	1037(55.6)	501(26.9)	192(10.3)	<.001	302(59.8)	174(34.5)	63(12.5)	<.001	735(54.1)	327(24.1)	129(9.58)	<.001	.06
Sweating	-	324(17.4)	20(1.1)	<.001	-	116(23.0)	3(0.6)	<.001	-	208(15.3)	17(1.3)	<.001	.22
Chest tightness	540(29.0)	243(13.0)	57(3.1)	<.001	181(35.8)	96(19.0)	23(4.6)	<.001	359(26.4)	147(10.8)	34(2.5)	<.001	.02
Anxiety	-	199(10.7)	81(4.3)	<.001	-	62(12.3)	35(6.9)	.004	-	137(10.1)	46(3.4)	<.001	.001
Myalgia	502(26.9)	145(7.8)	35(1.9)	<.001	138(27.3)	55(10.9)	14(2.8)	<.001	364(26.8)	90(6.6)	21(1.5)	<.001	.08
Palpitation	-	82(4.4)	8(0.4)	<.001	-	29(5.7)	2(0.4)	<.001	-	53(3.9)	6(0.4)	<.001	1.00[a]
Cough	1313(70.4)	72(3.9)	30(1.6)	<.001	377(74.7)	33(6.5)	9(1.8)	<.001	936(68.9)	39(2.9)	21(1.5)	.02	.72

© 2022 Yang X et al. *JAMA Network Open.*

Shortness of breath	781(41.9)	84(4.5)	18(1.0)	<.001	272(53.9)	34(6.7)	6(1.2)	<.001	509(37.5)	50(3.7)	12(0.9)	<.001	.60[a]
Dizziness	47(2.5)	66(3.5)	10(0.5)	<.001	14(2.8)	20(4.0)	4 (0.8)	.001	33(2.4)	46(3.4)	6(0.4)	<.001	.47[a]
Expectoration	312(16.7)	56(3.0)	24(1.3)	<.001	100(19.8)	25(5.0)	7(1.4)	.001	212(15.6)	31(2.3)	17(1.3)	.04	.82
Dyspnea	137(7.3)	49(2.6)	37(2.0)	.19	81(16.0)	23(4.6)	14(2.8)	0.13	56(4.1)	26(1.9)	23(1.7)	.67	.14
Headache	46(2.5)	42(2.3)	10(0.5)	<.001	7(1.4)	15(3.0)	1(0.2)	<.001	39(2.9)	27(2.0)	9(0.7)	.003	.30[a]
Edema of lower limbs	-	28(1.5)	6(0.3)	<.001	-	13(2.6)	2(0.4)	.004	-	15(1.1)	4(0.3)	.01	.67[a]
Taste change	-	26(1.4)	1(0.1)	<.001	-	10(2.0)	0	-	-	16(1.2)	1(0.1)	<.001	-
Smell reduction	-	25(1.3)	4(0.2)	<.001	-	12(2.4)	0	-	-	13(1.0)	4(0.3)	.03	-
Sore throat	110(5.9)	18(1.0)	2(0.1)	<.001	24(4.8)	10(2.0)	0	-	86(6.3)	8(0.6)	2(0.1)	.06	-
Anorexia	948(50.9)	13(0.7)	3(0.2)	<.001	264(52.3)	3(0.6)	1(0.2)	.62[a]	684(50.3)	10(0.7)	2(0.1)	.02	1.00[a]
Diarrhea	129(6.9)	13(0.7)	9(0.5)	.39	31(6.1)	4(0.8)	2(0.4)	.69[a]	98(7.2)	9(0.7)	7(0.5)	.62	1.00[a]
Hemoptysis	14(0.8)	2(0.1)	0	-	4(0.8)	0	0	-	10(0.7)	2(0.1)	0	-	-
Nausea	56(3.0)	3(0.2)	4(0.2)	1.00[a]	14(2.8)	0	0	-	42(3.1)	3(0.2)	4(0.3)	1.00[a]	-
Chill	43(2.3)	2(0.1)	1(0.1)	1.00[a]	21(4.2)	0	0	-	22(1.6)	2(0.1)	1(0.1)	1.00[a]	-
Vomiting	48(2.6)	1(0.1)	2(0.1)	1.00[a]	14(2.8)	0	0	-	34(2.5)	1(0.1)	2(0.1)	1.00[a]	-
Alopecia	-	-	15(0.8)	-	-	-	0	-	-	-	15(1.1)	-	-
Fever	1440(77.3)	-	-	-	410(81.2)	-	-	-	1030(75.8)	-	-	-	

a, Fisher's exact test. The rest of the statistical test were calculated with the Pearson χ^2 test. b, 1-year vs. 2-year

© 2022 Yang X et al. *JAMA Network Open.*

eTable 6. Characteristics of the Propensity Score-Matched Population

	Matched Follow-up	matched Lost to follow-up	*P* value
Numbers	1691	1691	
Age – Median (IQR), years[a]	60.0(50.0-68.0)	61.0(50.0-68.0)	.68
Sex – No. (%)			.27
Male	800(47.3)	832(49.2)	
Female	891(52.7)	859(50.8)	
Severe disease – No. (%)	425(25.1)	432(25.5)	.78
Cigarette smoking – No. (%)			.87
Never	1561(92.3)	1567(92.7)	
Former	26(1.5)	27(1.6)	
Active	104(6.2)	97(5.7)	
Coexisting disorders – No. (%)			
Coexisting disorders number			.08
0	959(56.7)	907(53.6)	
1	425(25.1)	482(28.5)	
≥2	307(18.2)	302(17.9)	
Hypertension	521(30.8)	522(30.9)	.97
Diabetes mellitus	236(14.0)	234(13.8)	.92
Cardiovascular diseases	155(9.2)	163(9.6)	.64
Chronic liver diseases	84(5.0)	91(5.4)	.59
Cerebrovascular diseases	39(2.3)	38(2.2)	.91
Chronic kidney diseases	35(2.1)	50(3.0)	.10
Tumor	30(1.8)	41(2.4)	.19
Tracheitis	23(1.4)	29(1.7)	.40
COPD	16(0.9)	23(1.4)	.26
Length of hospital stay, days [a]	14.0(9.0-21.0)	14.0(8.0-20.0)	.15
ICU admission – No. (%)	31(1.8)	26(1.5)	.50
Oxygen therapy, No. (%)	1181(69.8)	1225(72.4)	.10
Mechanical Ventilation, No. (%)	10(0.6)	9(0.5)	.82

Abbreviation: IQR, Inter-quartile range; ICU, Intensive Care Unit; COPD, Chronic obstructive pulmonary disease; NA, not applicable.

a, Mann-Whitney *U* test. The rest of the statistical test were calculated with the Pearson χ^2 test.

© 2022 Yang X et al. *JAMA Network Open.*

eTable 7. Symptoms at 1-Year Follow-up and 2-Year Follow-up in Overall and Propensity Score-Matched Populations

Symptoms	1-year			2-year		
	**Overall(n=1864)**	**Matched(n=1691)**	***P* value**	**Overall(n=1864)**	**Matched(n=1691)**	***P* value**
Any one of the following symptoms	806(43.2)	739(43.7)	.78	370(19.8)	338(20.0)	.92
Fatigue	501(26.9)	466(27.6)	.65	192(10.3)	178(10.5)	.83
Sweating	324(17.4)	297(17.6)	.89	20(1.1)	20(1.2)	.76
Chest tightness	243(13.0)	219(13.0)	.94	57(3.1)	47(2.8)	.62
Anxiety	199(10.7)	189(11.2)	.63	81(4.3)	74(4.4)	.96
Myalgia	145(7.8)	134(7.9)	.87	35(1.9)	32(1.9)	.97
Palpitation	82(4.4)	71(4.2)	.77	8(0.4)	6(0.4)	.72
Cough	72(3.9)	64(3.8)	.90	30(1.6)	28(1.7)	.91
Short of breath	84(4.5)	75(4.4)	.92	18(1.0)	15(0.9)	.81
Dizziness	66(3.5)	64(3.8)	.70	10(0.5)	7(0.4)	.60
Expectoration	56(3.0)	50(3.0)	.93	24(1.3)	24(1.4)	.73
Dyspnea	49(2.6)	45(2.7)	.95	37(2.0)	35(2.1)	.86
Headache	42(2.3)	42(2.5)	.65	10(0.5)	10(0.6)	.83
Edema of lower limbs	28(1.5)	25(1.5)	.95	6(0.3)	6(0.4)	.87
Taste change	26(1.4)	25(1.5)	.83	1(0.1)	1(0.1)	1.00[a]
Smell reduction	25(1.3)	24(1.4)	.84	4(0.2)	4(0.2)	1.00[a]
Sore throat	18(1.0)	14(0.8)	.66	2(0.1)	2(0.1)	1.00[a]
Anorexia	13(0.7)	12(0.7)	.97	3(0.2)	3(0.2)	1.00[a]
Diarrhea	13(0.7)	13(0.8)	.80	9(0.5)	8(0.5)	.97
Hemoptysis	2(0.1)	2(0.1)	1.00[a]	0	0	-
Nausea	3(0.2)	3(0.2)	1.00[a]	4(0.2)	4(0.2)	1.00[a]

© 2022 Yang X et al. *JAMA Network Open.*

Chill	2(0.1)	2(0.1)	1.00[a]	1(0.1)	1(0.1)	1.00[a]
Vomiting	1(0.1)	1(0.1)	1.00[a]	2(0.1)	2(0.1)	1.00[a]
Alopecia	-	-	-	15(0.8)	15(0.9)	.79

a, Fisher's exact test. The rest of the statistical test were calculated with the Pearson χ^2 test.

© 2022 Yang X et al. *JAMA Network Open.*

eTable 8. Symptoms at 2-Year Follow-up in Overall and Propensity Score-Matched Populations According to Disease Severity

Symptoms	Enrolled			Matched			*P* value (Enrolled vs matched)		
	**Enrolled**	**Severe**	**Non-severe**	**Enrolled**	**Severe**	**Non-severe**	**Enrolled**	**Severe**	**Non-severe**
Number	1864	505	1359	1691	425	1047			
Any one of the following symptoms	370(19.8)	123(24.4)	247(18.2)	338(20.0)	104(24.5)	234(18.5)	.92	.97	.84
Fatigue	192(10.3)	63(12.5)	129(9.5)	178(10.5)	55(12.9)	123(9.7)	.83	.83	.85
Sweating	20(1.1)	3(0.6)	17(1.3)	20(1.2)	3(0.7)	17(1.3)	.76	1.00[a]	.84
Chest tightness	57(3.1)	23(4.6)	34(2.5)	47(2.8)	16(3.8)	31(2.4)	.62	.55	.93
Anxiety	81(4.3)	35(6.9)	46(3.4)	74(4.4)	30(7.1)	44(3.5)	.96	.94	.90
Myalgia	35(1.9)	14(2.8)	21(1.5)	32(1.9)	12(2.8)	20(1.6)	.97	.96	.94
Palpitation	8(0.4)	2(0.4)	6(0.4)	6(0.4)	1(0.2)	5(0.4)	.72	1.00[a]	.85
Cough	30(1.6)	9(1.8)	21(1.5)	28(1.7)	8(1.9)	20(1.6)	.91	.91	.90
Short of breath	18(1.0)	6(1.2)	12(0.9)	15(0.9)	5(1.2)	10(0.8)	.81	.99	.79
Dizziness	10(0.5)	4 (0.8)	6(0.4)	7(0.4)	2 (0.5)	5(0.4)	.60	.69[a]	.85
Expectoration	24(1.3)	7(1.4)	17(1.3)	24(1.4)	7(1.6)	17(1.3)	.73	.75	.84
Dyspnea	37(2.0)	14(2.8)	23(1.7)	35(2.1)	12(2.8)	23(1.8)	.86	.96	.81
Headache	10(0.5)	1(0.2)	9(0.7)	10(0.6)	1(0.2)	9(0.7)	.83	1.00[a]	.88
Edema of lower limbs	6(0.3)	2(0.4)	4(0.3)	6(0.4)	2(0.5)	4(0.3)	.87	1.00[a]	1.00[a]
Taste change	1(0.1)	0	1(0.1)	1(0.1)	0	1(0.1)	1.00[a]	-	1.00[a]
Smell reduction	4(0.2)	0	4(0.3)	4(0.2)	0	4(0.3)	1.00[a]	-	1.00[a]
Sore throat	2(0.1)	0	2(0.1)	2(0.1)	0	2(0.2)	1.00[a]	-	1.00[a]
Anorexia	3(0.2)	1(0.2)	2(0.1)	3(0.2)	1(0.2)	2(0.2)	1.00[a]	1.00[a]	1.00[a]
Diarrhea	9(0.5)	2(0.4)	7(0.5)	8(0.5)	1(0.2)	7(0.6)	.97	1.00[a]	.89

© 2022 Yang X et al. *JAMA Network Open.*

Hemoptysis	0	0	0	0	0	0	-	-	-
Nausea	4(0.2)	0	4(0.3)	4(0.2)	0	4(0.3)	1.00[a]	-	1.00[a]
Chill	1(0.1)	0	1(0.1)	1(0.1)	0	1(0.1)	1.00[a]	-	1.00[a]
Vomiting	2(0.1)	0	2(0.1)	2(0.1)	0	2(0.2)	1.00[a]	-	1.00[a]
Alopecia	15(0.8)	0	15(1.1)	15(0.9)	0	15(1.2)	.79	-	.85

a, Fisher's exact test. The rest of the statistical test were calculated with the Pearson χ^2 test.

© 2022 Yang X et al. *JAMA Network Open.*

eTable 9. Symptom Dynamics of COVID-19 Survivors According to Disease Severity in Propensity Score-Matched Population

Categories	Number of symptoms		Number of Participants			*P* value
	1-y	2-y	Enrolled patients	Severe	Non-severe	Severe vs. Non-severe
Total			1691(100.0)	425(100.0)	1266(100.0)	
Symptom persist	≥1	≥1	207(12.2)	76(17.9)	131(10.3)	<.001
Symptom relief	≥1	0	532(31.5)	148(34.8)	384(30.3)	.08
Late-onset sequelae	0	≥1	131(7.7)	28(6.6)	103(8.1)	.30
No sequelae	0	0	821(48.6)	173(40.7)	648(51.2)	<.001

© 2022 Yang X et al. *JAMA Network Open.*

eTable 10. Logistic Regression Models to Evaluate the Risk Factors for Symptom Persist vs Relief

Variables	Univariable ORs(95%CI)	*P* value	Multivariable ORs(95%CI)	*P* value
Age, year	1.02(1.00-1.03)	.009	1.01(1.00-1.03)	.06
Sex, female	0.81(0.59-1.10)	.17	0.77(0.56-1.06)	.11
Severity, vs. no	1.53(1.11-2.11)	.009	1.37(0.97-1.91)	.07
ICU admission, vs. no	3.35(1.31-8.61)	.01	2.69(1.02-7.06)	.04
Oxygen therapy, vs. no	1.26(0.87-1.83)	.23		
Mechanical ventilation, vs. no	1.75(0.49-6.24)	.39		
Cigarette smoking, vs. never				
Former	0.87(0.23-3.26)	.84		
Active	1.08(0.56-2.06)	.83		
Coexisting disorder, vs. no				
Hypertension	1.31(0.95-1.81)	.10		
Diabetes	0.98(0.64-1.50)	.92		
Cardiovascular diseases	1.40(0.85-2.29)	.18		
Chronic liver diseases	1.51(0.82-2.80)	.19		
Cerebrovascular diseases	1.22(0.49-3.03)	.67		
Chronic kidney diseases	0.94(0.30-3.00)	.92		
Tumor	0.55(0.16-1.94)	.35		
Tracheitis	1.21(0.45-3.21)	.71		
COPD	0.52(0.06-4.45)	.55		

Dependent variable: symptom persist at 2-year follow-up.

Independent variables: age, sex, severity, ICU admission, and hypertension.

Abbreviations: ICU, intensive care unit; COPD, Chronic Obstructive Pulmonary Disease.

© 2022 Yang X et al. *JAMA Network Open.*

eTable 11. Logistic Regression Models to Evaluate the Risk Factors for Symptom New-Onset vs No Sequelae

Variables	Univariable ORs(95%CI)	*P* value	Multivariable ORs(95%CI)	*P* value
Age, year	1.02(1.00-1.03)	.02	1.01(1.00-1.03)	.07
Sex, female	0.82(0.58-1.16)	.26	0.81(0.57-1.15)	.24
Severity, vs. no	1.03(0.68-1.56)	.88	0.92(0.60-1.42)	.72
ICU admission, vs. no	1.81(0.59-5.57)	.30		
Oxygen therapy, vs. no	1.37(0.92-2.03)	.12		
Mechanical ventilation, vs. no	3.14(0.28-34.83)	.35		
Cigarette smoking，vs. never				
Former	2.01(0.72-5.61)	.19		
Active	0.58(0.25-1.39)	.22		
Coexisting disorder, vs. no				
Hypertension	1.26(0.86-1.85)	.24		
Diabetes	1.18(0.71-1.94)	.53		
Cardiovascular diseases	1.14(0.61-2.12)	.68		
Chronic liver diseases	1.04(0.46-2.37)	.92		
Cerebrovascular diseases	3.68(1.59-8.49)	.002	3.23(1.36-7.69)	.008
Chronic kidney diseases	1.76(0.64-4.82)	.27		
Tumor	1.57(0.44-5.64)	.49		
Tracheitis	0.62(0.08-4.90)	.65		
COPD	2.71(0.69-10.61)	.15		

Dependent variable: new-onset sequelae at 2-year follow-up.

Independent variables: age, sex, severity, and cerebrovascular diseases.

Abbreviations: ICU, intensive care unit; COPD, Chronic Obstructive Pulmonary Disease.

© 2022 Yang X et al. *JAMA Network Open.*

eTable 12. Logistic Regression Models to Evaluate the Risk Factors for More Than Two Symptoms at 2-Year Follow-up

Variables	Univariable ORs(95%CI)	*P* value	Multivariable ORs(95%CI)	*P* value
Age, year	1.03(1.01-1.04)	<.001	1.03(1.01-1.04)	<.001
Sex, female	0.79(0.56-1.12)	.19	0.77(0.54-1.09)	.14
Severity, vs. no	1.37(0.95-1.97)	.10	1.18(0.81-1.72)	.40
ICU admission, vs. no	0.71(0.17-2.98)	.64		
Oxygen therapy, vs. no	1.57(1.03-2.39)	.04		
Mechanical ventilation, vs. no	1.01(0.13-7.83)	.99		
Cigarette smoking，vs. never				
Former	1.68(0.58-4.88)	.34		
Active	0.64(0.27-1.50)	.30		
Coexisting disorder, vs. no				
Hypertension	1.14(0.79-1.64)	.50		
Diabetes	1.08(0.67-1.75)	.75		
Cardiovascular diseases	1.57(0.93-2.64)	.09		
Chronic liver diseases	1.99(1.08-3.67)	.03	2.22(1.19-4.14)	0.01
Cerebrovascular diseases	2.58(1.18-5.63)	.02		
Chronic kidney diseases	1.87(0.72-4.86)	.20		
Tumor	1.26(0.38-4.19)	.71		
Tracheitis	1.36(0.41-4.52)	.62		
COPD	0.81(0.11-6.16)	.84		

Dependent variable: more than two symptoms at 2-year follow-up.

Independent variables: age, sex, severity, Oxygen therapy (excluding mechanical ventilation), cardiovascular diseases, chronic liver diseases, and cerebrovascular diseases.

Abbreviations: ICU, intensive care unit; COPD, Chronic Obstructive Pulmonary Disease.

© 2022 Yang X et al. *JAMA Network Open.*

eTable 13. Patient Characteristics Between Those With CAT Total Scores ≥ 10 and Those < 10

	Enrolled patients (n=1864)	CAT≥10 (n=116)	CAT<10 (n=1748)	*P* value CAT≥10 vs. CAT<10
Age – Median (IQR), years[a]	58.5(49.0-68.0)	66.5(58.0-72.0)	58.0(48.0-67.0)	<.001
Sex – No. (%)				
Male	926(49.7)	59(50.9)	867(49.6)	.79
Female	938(50.3)	57(49.1)	881(50.4)	
Severe disease – No. (%)	505(27.1)	48(41.4)	457(26.1)	<.001
Cigarette smoking – No. (%)				.46[b]
Never	1711(91.8)	110(94.9)	1601(91.6)	
Former	33(1.8)	2(1.7)	31(1.8)	
Active	120(6.4)	4(3.4)	116(6.6)	
Coexisting disorders – No. (%)				
Coexisting disorders number				.01
0	1072(57.5)	54(46.6)	1018(58.2)	
1	464(24.9)	31(26.7)	433(24.8)	
≥2	328(17.6)	31(26.7)	297(17.0)	
Hypertension	544(29.2)	42(36.2)	502(28.7)	.09
Diabetes mellitus	259(13.9)	17(14.7)	242(13.8)	.80
Cardiovascular diseases	164(8.8)	13(11.2)	151(8.6)	.34
Chronic liver diseases	96(5.2)	11(9.5)	85(4.9)	.03
Cerebrovascular diseases	47(2.5)	4(3.4)	43(2.5)	.53[b]
Chronic kidney diseases	38(2.0)	5(4.3)	33(1.9)	.08[b]
Tumor	32(1.7)	5(4.3)	27(1.5)	.04[b]
Tracheitis	30(1.6)	2(1.7)	28(1.6)	.71[b]

© 2022 Yang X et al. *JAMA Network Open.*

COPD	16(0.9)	3(2.6)	13(0.7)	.07[b]
Length of hospital stay, days [a]	14.0(9.0-20.0)	16.0(11.0-23.0)	14.0(9.0-20.0)	.002
Follow-up time, days[a]	730(719-743)	730(717-743)	730(719-743)	.87
ICU administration – No. (%)	36(1.9)	8(6.9)	28(1.6)	.001[b]
Oxygen therapy, No. (%)	1341(71.9)	96(82.8)	1245(71.2)	.007
Mechanical Ventilation, No. (%)	14(0.8)	4(3.4)	10(0.6)	.009[b]

Abbreviation: IQR, Inter-quartile range; ICU, Intensive Care Unit; COPD, Chronic obstructive pulmonary disease; NA, not applicable.

a, Mann-Whitney U test; b, Fisher's exact test; The rest: Pearson $\chi 2$ test.

© 2022 Yang X et al. *JAMA Network Open.*

eTable 14. Logistic Regression Models to Evaluate the Risk Factors for CAT Score ≥10

Variables	Univariable ORs(95%CI)	*P* value	Multivariable ORs(95%CI)	*P* value
Age, year	1.05(1.03-1.06)	<.001	1.04(1.03-1.06)	<.001
Sex, female	1.05(0.72-1.53)	.79	1.02(0.69-1.50)	.92
Severity, vs. no	1.99(1.36-2.93)	<.001	1.41(0.93-2.13)	.11
ICU admission, vs. no	4.55(2.03-10.22)	<.001	2.83(1.21-6.66)	.02
Oxygen therapy, vs. no	1.94(1.19-3.17)	.008		
Mechanical ventilation, vs. no	4.61(1.25-17.00)	.02		
Cigarette smoking, vs. never				
Former	0.86(0.20-3.64)	.83		
Active	0.46(0.17-1.27)	.13		
Coexisting disorder, vs. no				
Hypertension	1.41(0.95-2.09)	.09		
Diabetes	1.07(0.63-1.82)	.81		
Cardiovascular diseases	1.34(0.73-2.43)	.35		
Chronic liver diseases	2.05(1.06-3.96)	.03	2.18(1.10-4.33)	.03
Cerebrovascular diseases	1.42(0.50-4.02)	.51		
Chronic kidney diseases	2.34(0.90-6.11)	.08		
Tumor	2.87(1.09-7.60)	.03		
Tracheitis	1.08(0.25-4.58)	.92		
COPD	3.54(1.00-12.61)	.05		

Dependent variable: CAT score ≥10.

Independent variables: age, sex, severity, ICU admission, oxygen therapy (excluding mechanical ventilation), Mechanical ventilation, hypertension, Chronic liver diseases, Chronic kidney diseases, tumor, and COPD.

Abbreviations: ICU, intensive care unit; COPD, Chronic Obstructive Pulmonary Disease.

© 2022 Yang X et al. *JAMA Network Open.*

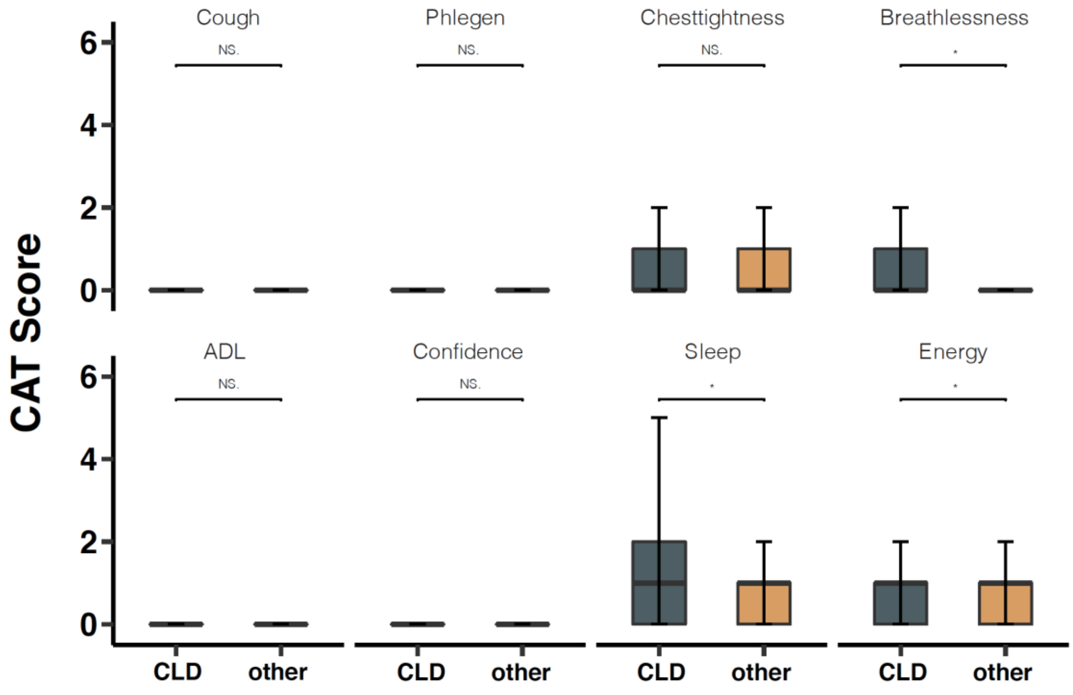

eFigure 2. CAT Item Scores Between Patients With and Without Coexisting Chronic Liver Disease

CLD: chronic liver disease. *, p<0.05.

© 2022 Yang X et al. *JAMA Network Open.*
